# Adolescent-parent disagreement on health-related quality of life in food allergic adolescents; who makes the difference?

**DOI:** 10.1186/2045-7022-1-S1-O40

**Published:** 2011-08-12

**Authors:** Jantina L van der Velde, Bertine MJ Flokstra-de Blok, Ann Hamp, Rebecca C Knibb, Eric J Duiverman, Anthony EJ Dubois

**Affiliations:** 1 University Medical Centre Groningen, University of Groningen, Pediatric Allergy and Pulmonology, Groningen, Netherlands; 2 University Medical Centre Groningen, University of Groningen, General practice, Groningen, Netherlands; 3 University of Derby, Department of Psychology, Derby, United Kingdom; 4 University Medical Centre Groningen, University of Groningen, Department of Pediatric Allergy and Pulmonology, Groningen, Netherlands

## Background

Food allergic adolescents are at highest risk for food allergy fatalities, which may be partly due to compromised self-management behaviour. Such behaviour may be negatively influenced by conflictual situations caused by child-parent disagreement on the adolescent’s Health-Related Quality of Life (HRQL). Comparisons of self- and parent-proxy-reported HRQL have never extensively been studied in food allergic adolescents. Therefore, the aims of this study were to investigate disagreement in self- and parent-proxy-reported HRQL of food allergic adolescents and to investigate the influence of participant characteristics, illness expectations and perceptions on adolescent-parent disagreement.

## Methods

Teenager Form (-TF) and -Parent Form (-PFA) of the Food Allergy Quality of Life Questionnaire (FAQLQ), Food Allergy Independent Measure (FAIM) and Brief-Illness Perception Questionnaire (Brief-IPQ) were sent to Dutch food allergic adolescents (13-17 years) and their parents. ICCs, t-tests and Bland-Altman plots were used to examine adolescent-parent agreement. Factors influencing agreement were studied (linear regression).

## Results

Seventy adolescent-parent pairs were included. There was a moderate correlation (ICC=0.61, p<0.001) and no significant difference (3.78 versus 3.56, p=0.136) between adolescent- and parent-proxy-reported HRQL. However, Bland-Altman plots showed relevant differences (exceeding minimal important difference) for 64% of all adolescent-parent pairs. Regression analysis showed that an older age of adolescents, poorer adolescent-reported illness comprehension (Brief-IPQ-TF, coherence) and higher adolescent-reported perceived disease severity (FAIM-TF) were associated with adolescent-parent disagreement on HRQL.

## Conclusions

Adolescent-parent agreement on HRQL was moderate. Adolescent-parent disagreement on HRQL was mainly determined by the adolescent’s rather than the parent's perceptions and characteristics. Illness comprehension may be an important target for intervention aimed at improving adolescent-parent agreement on HRQL. This may contribute to improved self-management of food allergic adolescents.

